# Maternal vitamin D status and risk of childhood overweight at 5 years of age in two Nordic cohort studies

**DOI:** 10.3389/fnut.2023.1201171

**Published:** 2023-07-26

**Authors:** Anna Amberntsson, Linnea Bärebring, Anna Winkvist, Lauren Lissner, Helle Margrete Meltzer, Anne Lise Brantsæter, Eleni Papadopoulou, Hanna Augustin

**Affiliations:** ^1^Department of Internal Medicine and Clinical Nutrition, Institute of Medicine, Sahlgrenska Academy, University of Gothenburg, Gothenburg, Sweden; ^2^School of Public Health and Community Medicine, Institute of Medicine, Sahlgrenska Academy, University of Gothenburg, Gothenburg, Sweden; ^3^Department of Food Safety, Division of Climate and Environmental Health, Norwegian Institute of Public Health, Oslo, Norway; ^4^Global Health Cluster, Division of Health Services, Norwegian Institute of Public Health, Oslo, Norway

**Keywords:** vitamin D, 25-hydroxyvitamin D, pregnancy, childhood overweight, childhood BMI, observational study, MoBa

## Abstract

**Introduction:**

Maternal vitamin D status during pregnancy has been suggested to have a role in childhood adiposity development, but results are conflicting. Our aims were to investigate [1] the relationships between maternal 25-hydroxyvitamin D (25OHD) during pregnancy and the child’s body mass index (BMI) and risk of overweight at 5 years of age, and [2] maternal pre-pregnancy BMI as effect modifier for these associations.

**Methods:**

Data sources included a subsample from the Norwegian Mother, Father and Child Cohort Study (MoBa sub-cohort; *N* = 2,744) and the Swedish GraviD cohort study (*N* = 891). Maternal 25OHD was analyzed in gestational week 18 in the MoBa sub-cohort and week 10 in the GraviD cohort. In the MoBa sub-cohort, parents reported their child’s documented measures of weight and length or height from the health card at routine check-up. In the GraviD cohort, this information was collected directly from medical records. Childhood overweight (including obesity) was identified using the International Obesity Task Force cut-offs. Linear and logistic regression models were used to investigate the association between maternal 25OHD and child’s BMI and risk of overweight at 5 years of age in each cohort separately, and in a pooled dataset.

**Results:**

In the pooled analysis, maternal 25OHD <30 nmol/L was associated with lower BMI in children at 5 years of age, but not with risk of overweight. Interaction analysis showed that the association was predominant among children of mothers with pre-pregnancy BMI ≥25 kg/m^2^.

**Conclusion:**

Low maternal vitamin D status, particularly in mothers with overweight or obesity, predicted lower BMI in their five-year-old children. However, there was no evidence of an effect on overweight in these children.

## 1. Introduction

According to global estimates from the World Health Organization (WHO), around 5.6% of all children under the age of 5 years had overweight or obesity in 2016 (1). The corresponding prevalence in children between 5 and 9 years of age was 20.6% (2). Childhood overweight or obesity is associated with earlier onset of some chronic metabolic disorders, such as type 2 diabetes (3). The risk of childhood obesity increases when the mother has pre-pregnancy obesity (4, 5), and the WHO emphasizes that pregnancy is a vulnerable period with profound implications for the child’s later health and development (6). Deficiency of certain micronutrients during pregnancy may have significant consequences on fetal development that lead to chronic diseases later in life (7, 8).

Vitamin D is a micronutrient essential for skeletal health (9). Vitamin D status is defined by plasma or serum concentrations of 25-hydroxyvitamin D (25OHD), and target threshold concentration is set to ≥50 nmol/L by the Nordic Nutrition Recommendations (10). Up to 28% of light-skinned pregnant women living in Nordic countries have a vitamin D status of <50 nmol/L. (11) However, there is a large seasonal variation in vitamin D status in the Nordic countries (12). Except for season, concentrations of 25OHD are also inversely associated with body size (13, 14), but the causality and biological mechanism has not been fully explained (15).

Maternal vitamin D status <50 nmol/L during pregnancy is associated with negative outcomes in the offspring, e.g., increased risk of rickets (9). Supplementation with vitamin D might lower the risk of pre-eclampsia, gestational diabetes, and low birthweight (16). Maternal vitamin D status in pregnancy has also been associated with childhood growth and adiposity (17). For example, we previously found that maternal 25OHD ≤75 nmol/L was associated with a higher body mass index (BMI) growth trajectory class during the first 2 years of life (18). Several other observational studies have investigated the association between maternal vitamin D status during pregnancy and different outcome measures of childhood adiposity, at various ages, but the results are inconclusive (19–22). Low maternal 25OHD has been associated with higher risk of overweight in 1-year-old infants, but not at 4 years of age (22) or with BMI at 5–6 years of age (21), whereas low maternal 25OHD was associated with increased adiposity at 4 and 6 years (19), and higher body fat percentage at 5–6 years of age (21). Maternal pre-pregnancy BMI has been identified as an effect modifier in some studies investigating the association between maternal vitamin D intake or status and childhood adiposity (19, 22, 23), but not all studies have considered this interaction. Maternal pre-pregnancy BMI might be a predisposing factor of childhood BMI and the risk of childhood obesity increases when the mother has obesity (5). As pre-pregnancy BMI is associated with both childhood BMI (5) and inversely associated with maternal 25OHD (13), and it is possible that pre-pregnancy BMI could modify the association between maternal vitamin D status and childhood BMI or overweight (19, 22). The combination of low maternal vitamin D status and high maternal BMI may have different implications compared with low maternal vitamin D status and normal maternal BMI.

In sum, although previous studies have investigated associations between maternal 25OHD during pregnancy and child BMI and overweight at different ages, it is still unclear whether maternal vitamin D status plays a role in the development of childhood adiposity. Furthermore, it is possible that maternal pre-pregnancy BMI modifies this association. In the present study, using two well-characterized Nordic pregnancy cohort studies, we aimed to [1] assess the association between maternal vitamin D status (25OHD) during pregnancy and the child’s BMI and risk of overweight at 5 years of age, and [2] investigate potential effect modification by maternal pre-pregnancy BMI on these associations.

## 2. Materials and methods

### 2.1. Study population

This study used a sub sample of mother–child pairs from The Norwegian Environmental Biobank (24) and the Swedish GraviD cohort study (12). The Norwegian Environmental Biobank is a sub-study established with the aim of biomonitoring nutrients and environmental contaminants in participants from The Norwegian Mother, Father and Child Cohort Study (MoBa) (25). The Norwegian Environmental Biobank sub-population included in the current study is henceforth referred to as the MoBa sub-cohort.

The current study was approved by the Regional Committee for Medical and Health Research Ethics in Northern Norway (REC 2019/770-12172). Establishment of the MoBa cohort and initial data collection were based on a license from the Norwegian Data Protection Agency and approval from the Regional Committees for Medical and Health Research Ethics in Norway. The MoBa cohort is currently subjected to regulations in the Norwegian Health Registry Act. The GraviD cohort was approved by the Regional Ethics Committee in Gothenburg and the Swedish Ethical Review Authority (897-11, T439-13, T085-14, 2019-05219). Written informed consent was obtained from all participating women and from both parents for the child’s participation. Both the MoBa sub-cohort and the GraviD cohort were conducted according to the Declaration of Helsinki. The current study is based on version 12 of the quality-assured MoBa data files released for research in January 2019.

MoBa is a population-based pregnancy cohort study conducted by the Norwegian Institute of Public Health (25). Participants were recruited from all over Norway from 1999 to 2008. Pregnant women were invited to participate through a postal invitation after they signed up for the routine ultrasound scan offered free of charge in their local hospital around gestational week 17–18. Forty-one percent of these women consented to participation in the study. Questionnaires were sent home to the mother during pregnancy and after delivery. At the time of data collection, the questionnaires were only available in Norwegian, but English translations of all questionnaires are available on the Norwegian Institute of Public Health website (26). Participants were asked to donate blood and urine samples at the time of the ultrasound examination. The MoBa cohort currently includes 114,500 children, 95,200 mothers, and 75,200 fathers. Women were eligible for inclusion in the Norwegian Environmental Biobank based on availability of blood and urine samples and genetic data from MoBa, as well as availability of the first six questionnaires and the father questionnaire (24). The Norwegian Environmental Biobank included 2,999 women, of whom 2,988 were eligible for the current study, i.e., women who were pregnant in 2002–2008, and had data on 25OHD analyzed in blood donated in pregnancy.

The GraviD cohort is a pregnancy cohort study that invited women attending antenatal care in 2013–2014 in parts of southwest Sweden (12). Study information was available in eight different languages. In total, 2,125 women were recruited in early pregnancy before completing gestational week 17. The families were then invited to participate in a follow-up of their child at 5 years of age, which included retrieval of the child’s medical records from birth.

For the current study, mother–child pairs from the MoBa sub-cohort and the GraviD cohort were included. Exclusion criteria were multiple births, malformations, and chromosomal anomalies. Inclusion criteria were the availability of data on 25OHD concentrations during pregnancy and at least one measurement of weight and height of the child after birth. The final study population consisted of 2,744 (92%) mother–child pairs in the MoBa sub-cohort and 891 (42%) mother–child pairs in the GraviD cohort (Supplementary Figure S1).

### 2.2. Data collection

For the MoBa sub-cohort, blood samples were obtained from both parents around gestational week 18. Three questionnaires were sent out to the mother’s during pregnancy (Q1-Q3). The questionnaires at gestational weeks 17 (Q1) and 30 (Q3) included background information, such as weight, height, education level, and country of birth, while a food frequency questionnaire was answered in gestational week 22 (Q2). Vitamin D supplementation is not routinely recommended to pregnant women in Norway, except for women who eat fatty fish less than 2–3 times/week (27, 28). Birth weight and length were retrieved from the Medical Birth Registry, a national health registry containing information about all births in Norway (29). After delivery, the mother received questionnaires when the child was 6 months (Q4), 18 months (Q5), 3 years (Q6), 5 years (Q-5 year), 7 years (Q-7 year), and 8 years old (Q-8 year). The postnatal questionnaires asked the parents to report weight and length or height measurements of the child at 11 ages; 6 weeks, 3, 6, 12, and 18 months, 2, 3, 5, 7, and 8 years. The postnatal questionnaires asked the parents to report weight and length or height measurements of the child at 11 ages; 6 weeks, 3, 6, 12, and 18 months, 2, 3, 5, 7, and 8 years. From 0 to 5 years, the measures were assessed by public health nurses and reported in the child’s health card during routine check-up. On average, there were nine measurements of weight and height per child. Less than 1% of the children in the MoBa sub-cohort had only one measurement of weight and height during childhood. Retention rates of the reported measurements in the MoBa sub-cohort are provided in Supplementary Table S1.

**Figure 1:**
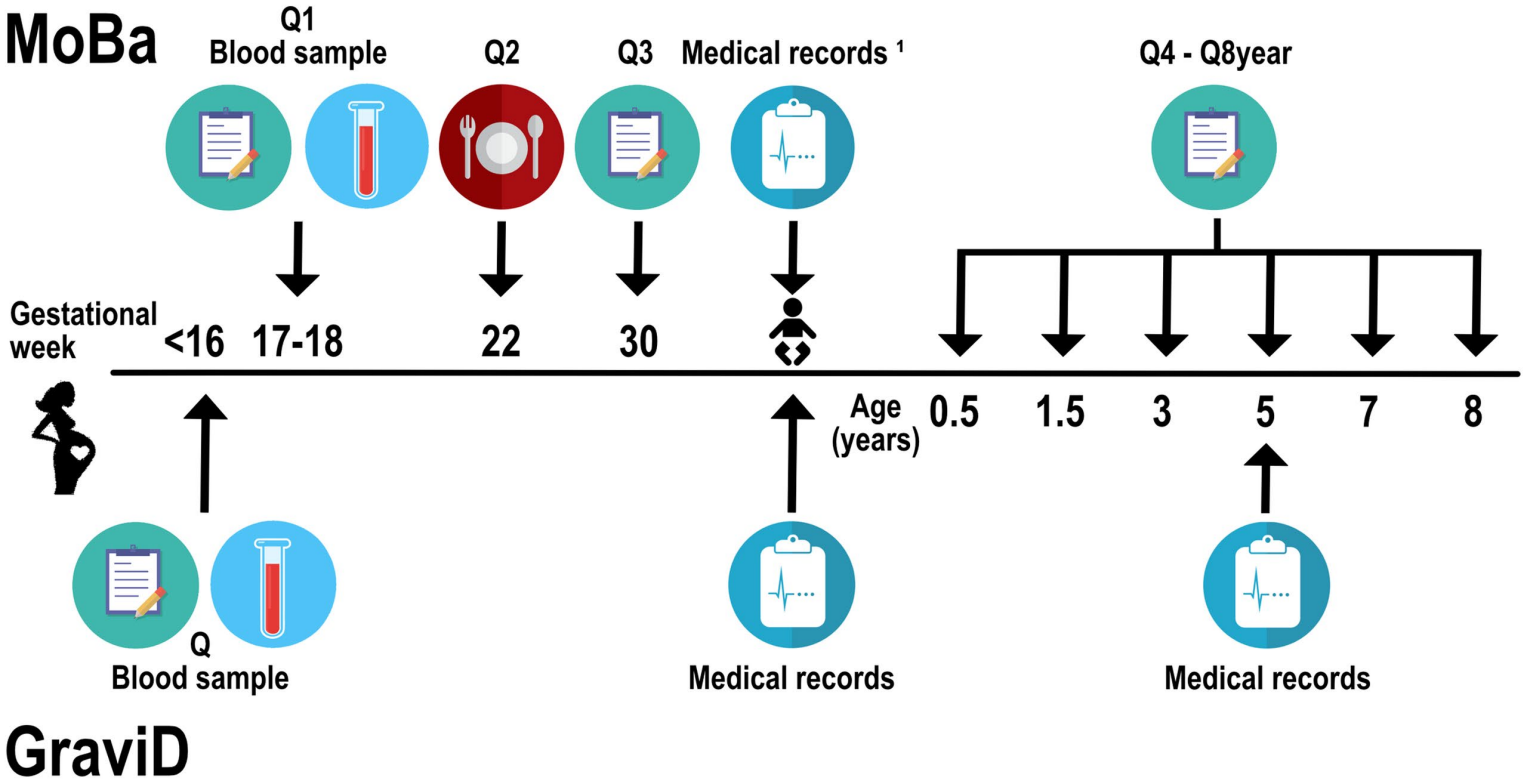
Flow chart for collection of data used in the current study, including the MoBa sub-cohort and GraviD cohort. Q, questionnaire. MoBa, the Norwegian Mother, Father and Child Cohort Study. ^1^Standard variables retrieved from the child’s birth records in the Medical Birth Registry of Norway.

In the GraviD cohort, women were recruited during two time-periods: September 2nd-November 8th in 2013, and February 24th-June 13th in 2014. Blood samples were collected twice from each participant at the antenatal care clinic, i.e., once before gestational week 17 and once after gestational week 31. During the first visit, the women reported background information, e.g., weight, height, education level, and country of birth, in a questionnaire. Pregnant women in Sweden are not routinely given vitamin D supplements during pregnancy. The midwives give advice according to the general recommendations (to those who do not eat vitamin D rich foods, or those with little sun exposure) (30). After delivery, medical records from antenatal care and obstetric units were collected where complementary data were obtained on, e.g., tobacco use and pre-pregnancy BMI. At 5 years of age, the child’s medical record was retrieved, providing multiple measurements of weight and height since birth assessed by public health nurses, as well as information on the child’s health status. In Sweden, weight and height are measured as part of routine child health care within 2 weeks after birth, 2–8 weeks, 3–5 months, 6, 8, 10, 12, and 18 months, 3, 4 and 5 years of age with an average of 16 measurements of weight and height per child. Around 5% of the children in the GraviD study had only one measurement of weight and height during childhood. An overview of the collection of data used in the current study in MoBa and GraviD is provided in Figure 1.

#### 2.2.1. Blood sample analysis

Blood sampling was only performed in autumn and spring in the GraviD cohort, while in the MoBa sub-cohort, blood samples were taken throughout the year. In the MoBa sub-cohort, vitamin D status was assessed by analyzing 25OHD in plasma using the Architect ci8200 system (Abbott Laboratories, Abbott Park, IL, United States) in 2015. The method is a high through-put automated chemiluminescent microparticle immunoassay. Analyzes were conducted at the National Institute for Health and Welfare in Helsinki, Finland. In the GraviD cohort, 25OHD was analyzed in serum using liquid chromatography tandem mass spectrometry (LC–MS/MS) at the central laboratory at the University Hospital in Malmö (31). Both assay methods analyzed 25OHD3 and 25OHD2, and the sum of the two metabolites was considered as the 25OHD. The laboratories were certified by the vitamin D External Quality Assessment Scheme (32). Further details on the blood sample analysis in the GraviD cohort has been described elsewhere (12). The blood sample drawn before gestational week 17 was used in the GraviD cohort. Additionally, the vitamin D Standardization Program protocol, compensating for assay variability, was applied in the GraviD study as previously described (18).

### 2.3. Growth modeling and definition of overweight

The Jenss-Bayley growth curve model (33) was used to obtain individual weight and height growth trajectories until 5 years of age. The growth curve model was used through a mixed effects approach by adding individual random effects in each equation parameter and fitted using Stochastic Approximation of Expectation–Maximization algorithm. All available reported anthropometric measurements for each child were used to predict the child’s growth trajectory. Implausible reported anthropometric values were identified and excluded by identifying measured values with >3 SD difference from the predicted value. In total, 463 (1.2%) weight and 215 (0.6%) height measurements were excluded. The correlation between measured and predicted values ranged from 0.94 to 0.99 for both weight and height. Individual weight and height were then predicted on the exact day each child turned 5 years old, using only the remaining values. BMI was then calculated as weight (kg) divided by squared height (m). Children were classified as having normal weight or overweight (including obesity) according to the International Obesity Task Force cut-offs (34).

### 2.4. Statistical analysis

Differences in study population characteristics were assessed using independent samples t-test for normally distributed variables, Wilcoxon-Mann Whitney test for ordinal variables, and Chi-square test for categorical variables. Significance was accepted at *p* < 0.05 and *p* < 0.2 for interaction terms. STATA version 16 was used for all statistical analyzes (Stata Corporation, College Station, Texas) and RStudio version 3.4.2 was used for the growth models.

In each cohort, separate linear and logistic regression models were used to investigate maternal 25OHD in relation to child’s BMI and risk of overweight at 5 years of age, respectively. Mixed effects linear and logistic regression models, with cohort as a random effect, were then used to investigate the same associations in a pooled dataset of both cohorts. The associations between maternal 25OHD and child’s BMI and risk of overweight in each cohort were also investigated using restricted cubic splines to test for non-linearity. The splines were modeled with five knots positioned at percentiles 5, 27.5, 50, 72.5 and 95% as recommended by Harrell (35). *p*-values for the splines model are reported for the overall non-linear associations between the exposure variable and the outcomes by testing the coefficients of all spline transformations equal to zero. By testing the coefficients of the second and third spline transformations equal to zero, non-linearity was investigated.

Maternal 25OHD was investigated as a categorical variable (>75 nmol/L reference, 50–75 nmol/L, 30–49.9 nmol/L, and < 30 nmol/L). A 25OHD ≥50 nmol/L has been suggested by the Institute of Medicine to promote bone health (36), while 25OHD ≥75 nmol/L has been proposed to promote overall health (37). A 25OHD concentration < 30 nmol/L has been suggested to define vitamin D deficiency (10, 36). Based on prior knowledge and informed by a directed acyclic graph (Supplementary Figure S2), covariates were selected. The models are presented as (1) crude, (2) minimally adjusted, including maternal education (<13, 13–16, >16 years), country of origin in respective cohort (Norwegian/Swedish or other), pre-pregnancy BMI (<18.5–24.9, 25–29.9, ≥30 kg/m^2^) and child’s sex, and (3) fully adjusted model, including the variables from model 2 plus maternal age (continuous), smoking during pregnancy (dichotomized), and parity (nulliparous or parous). Variables included in the fully adjusted model were the variables identified as confounders. Additionally, child’s sex was included *a priori*, as child’s sex is well-known to affect childhood BMI. Although no multicollinearity was detected between the covariates, some variables might still be related to each other and cause overadjustment in the model when all these variables are included. Thus, a model with fewer variables is presented (minimally adjusted) to be transparent with the effect of including all the covariates in the model.

Season of blood sampling was also included as a covariate in the fully adjusted model. Although it is not considered a confounder, it was included to account for other possible factors associated with season, and to compensate for the difference in season of blood sampling between the cohorts.

Maternal pre-pregnancy BMI (categorical) was investigated as an effect modifier in the pooled dataset, based on the association between maternal pre-pregnancy BMI and later child BMI (5), and on previous literature (19, 22, 23).

## 3. Results

### 3.1. Study population

The mean (SD) gestational week for analysis of maternal 25OHD was 18.3 (1.3) in the MoBa sub-cohort and 10.7 (1.8) in the GraviD cohort. A significantly larger proportion of the women in the MoBa sub-cohort were born in Norway, compared with the proportion of women in the GraviD cohort born in Sweden (Table 1). The MoBa sub-cohort had a higher proportion of women who smoked during pregnancy and were younger than women in the GraviD cohort. However, the neonates in the MoBa sub-cohort had higher birth weights and were breastfed for a longer period compared with the neonates in the GraviD study. At 5 years of age, the children in the MoBa sub-cohort had a higher BMI, and a larger proportion of the children were classified as having overweight or obesity, compared with the children in the GraviD cohort. There were no significant differences between the study populations in maternal educational level or pre-pregnancy BMI.

**Table 1:** Study population characteristics by cohort.

Variable	MoBa sub-cohort (*N* = 2,744)	GraviD cohort (*N* = 891)	
	*N* (%)	*N* (%)	*p*-value
Maternal education (years)			0.081
<13	728 (26.5)	192 (21.5)	
13–16	1,315 (47.9)	473 (53.1)	
>16	701 (25.6)	202 (25.4)	
Born in Norway or Sweden^a^			<0.001
Yes	2,579 (94.0)	758 (85.1)	
Pre-pregnancy BMI (kg/m^2^)			0.617
<18.5	79 (2.9)	21 (2.4)	
18.5–24.9	1,785 (64.6)	569 (63.8)	
25–29.9	669 (24.8)	231 (25.9)	
≥30	211 (7.7)	70 (7.9)	
Maternal smoking in pregnancy			0.016
Yes	154 (5.6)	28 (3.1)	
Parity			<0.001
Nulliparous	1,417 (51.6)	405 (45.5)	
Maternal 25OHD (nmol/L)			<0.001
<30	295 (10.8)	48 (5.4)	
30–49.9	1,020 (37.2)	200 (22.4)	
50–75	1,133 (41.3)	491 (55.1)	
>75	296 (10.8)	152 (17.1)	
Season of blood sampling			<0.001
November–April	1,481 (54.0)	322 (36.1)	
May–October	1,263 (46.0)	569 (63.9)	
Maternal vitamin D supplement use (yes)	2,218 (80.8)	419 (47.0)	<0.001
Child overweight at 5 years of age^b^	544 (19.8)	77 (8.6)	<0.001
	Median (p25-p75)	Median (p25-p75)	
Maternal 25OHD (nmol/L)	51.0 (39.0–64.0)	60.1 (49.0–70.4)	<0.001
Maternal age (years)	30 (27–33)	32 (29–35)	<0.001
Gestational age (days)	282 (275–287)	281 (275–287)	0.774
Birth weight (g)	3,650 (3,336-3,970)	3,565 (3,248-3,885)	<0.001
Lactation (months)	10 (7–13)	8 (6–12)	<0.001
Child BMI at 5 years of age	16.1 (15.3–17.0)	15.6 (14.7–16.3)	<0.001

25OHD, 25-hydroxyvitamin D; BMI, Body Mass Index; p25, 25th percentile; p75, 75th percentile. Statistical difference between the MoBa sub-cohort and GraviD cohort using independent samples t-test of normally distributed variables, Wilcoxon-Mann–Whitney test of non-normally distributed variables and Chi2 test of categorical variables. ^a^Born in the country of the cohort. ^b^Normal weight or overweight (including obesity) were classified according to the International Obesity Task Force (34) using predicted BMI.

### 3.2. Maternal 25OHD and child’s BMI at 5 years of age

Maternal 25OHD <30 nmol/L was associated with lower child’s BMI at 5 years of age in the fully adjusted model of the pooled dataset (Table 2). When season of blood sampling (November–April, May–October) was included in the fully adjusted model of the pooled dataset, the association became non-significant (50-75 nmol/L: β −0.05, 95% CI: −0.19, 0.09, *p* = 0.461; 30–49.9 nmol/L: β 0.01, 95% CI: −0.14, 0.16, *p* = 0.916; <30 nmol/L: β −0.19, 95% CI: −0.38, 0.01, *p* = 0.064). No association was found in the non-linear model using restricted cubic splines (Supplementary Figure S3).

**Table 2:** The association between maternal 25OHD during pregnancy and child’s BMI (kg/m^2^) at 5 years of age.

	MoBa sub-cohort (*N* = 2,744)			GraviD cohort (*N* = 891)			Pooled ^c^ (*N* = 3,635)		
	*β*	95% CI	*P*-value	*β*	95% CI	*P*-value	*β*	95% CI	*P*-value
Crude									
>75 nmol/L (reference)									
50–75 nmol/L	0.07	−0.11, 0.25	0.432	−0.15	−0.38, 0.09	0.225	−0.00	−0.14, 0.14	0.989
30–49.9 nmol/L	0.18	0.00, 0.36	0.047	−0.01	−0.29, 0.27	0.941	−0.12	−0.03, 0.27	0.112
<30 nmol/L	0.06	−0.16, 0.29	0.580	−0.21	−0.63, 0.22	0.338	−0.01	−0.21, 0.19	0.929
Minimally adjusted^a^									
>75 nmol/L (reference)									
50–75 nmol/L	0.00	−0.17, 0.18	0.980	−0.17	−0.40, 0.07	0.162	−0.05	−0.19, 0.09	0.495
30–49.9 nmol/L	0.07	−0.11, 0.25	0.454	−0.11	−0.38, 0.17	0.448	0.02	−0.13, 0.17	0.808
<30 nmol/L	−0.12	−0.34, 0.10	0.294	−0.34	−0.78, 0.10	0.132	−0.17	−0.36, 0.03	0.090
Fully adjusted^b^									
>75 nmol/L (reference)									
50–75 nmol/L	−0.01	−0.18, 0.17	0.955	−0.17	−0.40, 0.06	0.154	−0.06	−0.20, 0.08	0.428
30–49.9 nmol/L	0.05	−0.13, 0.23	0.607	−0.12	−0.40, 0.15	0.380	−0.00	−0.15, 0.15	0.980
<30 nmol/L	−0.15	−0.38, 0.07	0.180	−0.39	−0.83, 0.06	0.087	−0.20	−0.39, −0.01	0.043

25OHD, 25-hydroxyvitamin D; β, beta coefficient; CI, confidence interval; BMI, Body Mass Index. ^a^Including maternal education, country of origin, pre-pregnancy BMI, and child’s sex in a linear regression model. ^b^Including maternal education, country of origin, pre-pregnancy BMI, child’s sex, maternal age, smoking during pregnancy, and parity in a linear regression model. ^c^Mixed effects linear regression model with cohort as random effect in a pooled dataset.

Maternal pre-pregnancy BMI modified the association (*p* = 0.041) between maternal 25OHD and the child’s BMI at 5 years of age in the pooled dataset (Supplementary Figure S4). Lower maternal 25OHD was associated with lower child’s BMI at 5 years of age only in children of mothers with pre-pregnancy overweight or obesity.

### 3.3. Maternal 25OHD and risk of childhood overweight at 5 years of age

The proportion of children classified as having overweight (including obesity) by each category of maternal pre-pregnancy BMI was lower in the GraviD cohort, compared with the MoBa sub-cohort (Table 3).

**Table 3:** The prevalence of overweight at 5 years of age by category of maternal pre-pregnancy BMI in the MoBa sub-cohort and the GraviD cohort.

Maternal pre-pregnancy BMI (kg/m^2^)	Child having normal weight at 5 years of age^a^ *N* (%)	Child having overweight at 5 years of age^a^ *N* (%)
MoBa sub-cohort (*N* = 2,744)		
<25	1,586 (85.1)	278 (14.9)
25–29.9	481 (71.9)	188 (28.1)
≥30	135 (64.0)	76 (36.0)
GraviD cohort (*N* = 891)		
<25	557 (94.4)	33 (5.6)
25–29.9	201 (87.0)	30 (13.0)
≥30	56 (80.0)	14 (20.0)

BMI, Body Mass Index. ^a^Normal weight or overweight (including obesity) were classified according to the International Obesity Task Force (34) using predicted BMI.

There was an association between maternal 25OHD 30–49.9 nmol/L and a higher risk of childhood overweight at 5 years of age compared with maternal 25OHD >75 nmol/L in the crude model in the MoBa sub-cohort (OR 1.63, 95% CI 1.15–2.32) and in the pooled dataset (OR 1.46, 95% CI 1.07–2.00; Table 4). After adjustment, these associations were attenuated and no longer statistically significant. In the GraviD cohort, no association was found between maternal 25OHD and the child’s risk of overweight at 5 years of age. Including season of blood sampling (November–April, May–October) in the fully adjusted models did not change the estimates (data not shown). In the continuous non-linear model using restricted cubic splines, maternal 25OHD was not associated with the child’s risk of overweight at 5 years of age in any cohort (Supplementary Figure S5).

**Table 4:** The association between maternal 25OHD during pregnancy and the child’s risk of overweight at 5 years of age.

	MoBa sub-cohort (*N* = 2,744)			GraviD cohort (*N* = 891)			Pooled^c^ (*N* = 3,312)		
	OR	95% CI	*P*-value	OR	95% CI	*P*-value	OR	95% CI	*P*-value
Crude									
>75 nmol/L (reference)	1.0			1.0			1.0		
50–75 nmol/L	1.34	0.94, 1.91	0.103	0.70	0.37, 1.32	0.272	1.17	0.86, 1.59	0.318
30–49.9 nmol/L	1.63	1.15, 2.32	0.007	0.96	0.47, 1.95	0.980	1.46	1.07, 2.00	0.016
<30 nmol/L	1.37	0.89, 2.11	0.151	1.83	0.72, 4.62	0.203	1.34	0.91, 1.97	0.141
Minimally adjusted^a^									
>75 nmol/L (reference)	1.0			1.0			1.0		
50–75 nmol/L	1.17	0.81, 1.67	0.404	0.65	0.34, 1.24	0.190	1.04	0.76, 1.41	0.828
30–49.9 nmol/L	1.32	0.92, 1.89	0.130	0.77	0.37, 1.61	0.487	1.19	0.87, 1.64	0.279
<30 nmol/L	0.97	0.62, 1.52	0.906	1.45	0.51, 4.13	0.487	0.96	0.64, 1.43	0.825
Fully adjusted^b^									
>75 nmol/L (reference)	1.0			1.0			1.0		
50–75 nmol/L	1.18	0.82, 1.69	0.379	0.66	0.34, 1.27	0.212	1.04	0.76, 1.42	0.815
30–49.9 nmol/L	1.32	0.91, 1.89	0.140	0.73	0.35, 1.54	0.416	1.18	0.86, 1.63	0.307
<30 nmol/L	0.97	0.62, 1.52	0.894	1.27	0.44, 3.71	0.658	0.95	0.63, 1.42	0.802

25OHD, 25-hydroxyvitamin D; CI, Confidence Interval; OR, Odds Ratio. ^a^Including maternal education, country of origin, pre-pregnancy BMI, and child’s sex in a logistic regression model. ^b^Including maternal education, country of origin, pre-pregnancy BMI, child’s sex, maternal age, smoking during pregnancy, and parity in a logistic regression model. ^c^Mixed effects logistic regression model with cohort as random effect in a pooled dataset.

In the pooled dataset, maternal pre-pregnancy BMI was an effect modifier (*p* = 0.113) of the association between maternal 25OHD and the child’s risk of overweight at 5 years of age. However, after stratifying the analysis by maternal pre-pregnancy BMI, no significant associations were found (Supplementary Figure S6).

## 4. Discussion

In the current study, we investigated the relationship between maternal vitamin D status during pregnancy and childhood overweight. We found an association between maternal 25OHD <30 nmol/L and lower child BMI at 5 years of age in the pooled analysis. Additionally, we found indications that maternal pre-pregnancy BMI was an effect modifier. The association between lower maternal vitamin D status and lower child BMI at 5 years of age was only found in children of mothers with pre-pregnancy overweight or obesity. However, we found no consistent indication that lower maternal vitamin D status was associated with lower child BMI throughout the maternal vitamin D status categories. Also, no association between maternal vitamin D status and child’s risk of overweight was found.

There has been controversy as to whether maternal vitamin D status has implications for the risk of offspring adiposity. We have previously seen that lower maternal 25OHD was associated with a higher BMI growth trajectory class during the first 2 years of life (18), possibly affecting the child’s health beyond infancy. The novelty of the current paper includes investigation of the association between maternal 25OHD and the child’s BMI and overweight at 5 years of age. In line with the results from the current study, no association was observed between maternal 25OHD3 in mean gestational week 14 with BMI or overweight in children at 4 years of age in a Spanish population (22). In a Dutch population study, there was no association between maternal 25OHD in gestational week 16 and the child’s BMI at 5–6 years of age, and no effect modification with pre-pregnancy BMI observed (21). In contrast to our results, a study in a Greek population found a significantly higher BMI z-score at 4 and 6 years of age in the children of mothers with a 25OHD <37.7 nmol/L compared with ≥37.7 nmol/L in gestational week 14 (19). Since their cut-off was higher compared to the lowest maternal vitamin D status category in the current study, comparison between the studies is not straightforward. The vitamin D status might reflect overall health as it is an aggregation of time spent outdoors and intake of nutrient dense foods. Thus, it is plausible that residual confounding is present in our study. Additionally, 25OHD has also been suggested to be an acute phase protein (38), which then may introduce exposure misclassification in our study. It is possible that 25OHD might reflect conditions other than vitamin D status, which also might contribute to inconsistent results between studies.

We found that the association between lower maternal vitamin D status and lower child BMI at 5 years of age was only found in children of mothers with pre-pregnancy overweight or obesity. In concordance to our results, the Greek study reported maternal pre-pregnancy BMI to be an effect modifier (19). Children of women with pre-pregnancy BMI <30 kg/m^2^ and 25OHD <37.7 nmol/L had higher BMI z-scores, compared with ≥37.7 nmol/L, whereas children of mothers with pre-pregnancy obesity and 25OHD <37.7 nmol/L had lower BMI z-scores at 4 and 6 years of age. A possible explanation for this finding could be that women with pre-pregnancy obesity and 25OHD concentration < 30 nmol/L might differ from those with pre-pregnancy obesity and 25OHD concentration > 75 nmol/L in terms of nutrition status, weight status, and/or gestational weight gain. In addition, both the reference maternal vitamin D category (>75 nmol/L) and the category where the association was found (<30 nmol/L) contains few women among those with pre-pregnancy overweight or obesity. The results might also be explained by residual confounding. The biological mechanism and the importance of this finding remains unclear.

In addition to different categorizations of maternal vitamin D status, inconsistent results between studies might also be explained by definition of the outcomes. As the present recommendation for vitamin D status is set to maintain bone health, the optimal maternal 25OHD concentration for childhood health is unknown and different cut-offs are used in different studies. Possibly, additional measures beyond BMI and classification of overweight, such as body composition and other cardiometabolic risk markers, needs to be investigated to fully assess the role of maternal vitamin D status for childhood adiposity and health (21). As a proxy for adiposity, BMI might have low sensitivity in a child population (39) and should be accompanied by other measures for robustness. The time point of maternal blood sampling also differs between studies, which may be of importance as there could be different windows of sensitivity of maternal vitamin D status on the fetus. Also, the time point of investigation of the child’s anthropometry is likely of great importance. Adiposity rebound commonly occurs around 4–6 years of age (40) when there can be a large difference in growth and body composition between children. As a result, some children might be incorrectly classified as having overweight.

We have previously found a seasonal variation in vitamin D status in pregnant women in the Nordic countries (12). However, according to the directed acyclic graph (Supplementary Figure S2), season of blood sampling was not a confounder but rather a determinant of 25OHD concentration. Therefore, season of blood sampling was not included in the main models. However, after including season in the model with child BMI, the value of p became non-significant in the pooled dataset. Including season only slightly attenuated the estimates, which might be explained by the weak association between season of blood sampling and childhood BMI.

Intra uterine exposure to low vitamin D status during pregnancy might affect the adiposity of the child through fetal programming (9). A potential modulating role of vitamin D in adipose tissue inflammation has been supported, possibly affecting the adipogenesis of the fetus (41). Other research suggests an association between maternal vitamin D deficiency and increased maternal insulin resistance (42), exposing the fetus to elevated glucose levels and thereby an increased fetal insulin production and risk of later metabolic disturbances (43). Alternatively, exposure to vitamin D deficiency *in utero* may impact offspring adiposity development through an inflammatory pathway (44). However, the mechanism of the suggested relationship between maternal vitamin D status and the child’s adiposity is unknown.

### 4.1. Strengths and limitations

The current study benefits from being comprised of two Nordic pregnancy cohort studies, allowing for a detailed investigation of the association between maternal 25OHD and child’s BMI and risk of overweight at 5 years of age. Both cohorts have a long follow-up and a large study population. In the GraviD cohort, 25OHD was standardized according to the Vitamin D Standardization Program. The prediction of child’s BMI at 5 years of age using the Jenss-Bayley growth curve model seeks to balance selection bias related to loss to follow-up in both cohorts.

However, the study is not without limitations, including selection biases which may differ between studies. In GraviD, less than half of the women included during pregnancy in the original cohort were included in this study due to loss to follow-up from pregnancy to 5 years post-partum. Although the original GraviD cohort reflects the average pregnant population in Sweden (12), it is possible that our current study sample is not representative, and that the generalizability is impaired. In the MoBa sub-cohort, the eligible women were those with good compliance in the original protocol, as it required available data from three questionnaires answered during pregnancy, the father questionnaire, and the first three postnatal questionnaires. The selection bias may weaken the generalizability of this subsample. However, the selection bias in the whole MoBa cohort has been described previously and suggests the estimates for associations between exposures and outcomes are unbiased (45). The quality of the measurements of weight and height might also differ between the cohorts. In the GraviD cohort, more measurements of weight and height per child were available compared with the MoBa sub-cohort. In addition, the measurements were obtained from medical records in the GraviD cohort, while in the MoBa sub-cohort the anthropometrics were reported from the parents but instructed to be copied from the child’s health card. The difference in proportion of children classified as overweight between the cohorts might partially reflect this. The self-report of maternal background characteristics might be prone to misreporting and is also a limitation. Despite the benefits of the growth model, the values of BMI used to classify overweight were predictions, and not the measured values of weight and height. The predictions can have led to misclassification of some children, perhaps especially in those with few anthropometric measurements. Additionally, blood sampling was only performed in autumn and spring in the GraviD cohort, while in the MoBa sub-cohort samples were taken throughout the year. Thus, seasonal variations in 25OHD might also have affected the results. Further, blood sampling was performed in the first trimester in the GraviD cohort but in the second trimester in the MoBa sub-cohort, which may have also contributed to the difference in results. In addition, different assay methods and laboratories have been used for the analysis of 25OHD in the two cohorts, which can have contributed to the differences in median concentrations. Underestimation of 25OHD using Abbott Architect chemiluminescence immunoassay compared with LC–MS/MS has been found in some studies (46–48). Standardization of 25OHD in MoBa was not possible as there was no plasma left for such analysis. If the 25OHD concentrations in MoBa was underestimated, this could potentially lead to biased estimates and less relevant categories of exposure.

### 4.2. Implications

The association between maternal vitamin D status <30 nmol/L and lower child BMI at 5 years of age was only found in children of mothers with pre-pregnancy overweight or obesity and not in mothers with pre-pregnancy normal weight. In addition, we found no trend that lower maternal vitamin D status throughout the categories was associated with lower child BMI. Also, the results were not supported by an association between maternal vitamin D status and child’s risk of overweight. Thus, this study adds to existing evidence for a weak or non-existent relationship between maternal vitamin D status during pregnancy and the child’s BMI and overweight at early childhood. Further research should focus on different types of childhood outcomes reflecting adiposity, such as body composition, and risk markers of cardiometabolic health to fully assess whether childhood health should be added to the list of effects of maternal vitamin D status. Future studies could also focus on the cut-offs for vitamin D status where previous evidence for an association have been found. Evidently, vitamin D status should also be assessed along with the maternal pre-pregnancy BMI due to its effect modifying capacity.

### 4.3. Conclusion

In this study of two Nordic pregnancy cohorts, low maternal vitamin D status, particularly in mothers with overweight or obesity, predicted lower BMI in their five-year-old children. However, there was no evidence of an effect on overweight in these children.

## Data availability statement

Data from the MoBa sub-cohort and the GraviD cohort cannot be publicly shared. The consent given by the participants in the cohorts does not open for storage of data on an individual level in repositories or journals. The data contains potentially identifying or sensitive patient information. Data from MoBa used in this study are owned and managed by a third-party organization, the national health register holders in Norway (Norwegian Institute of Public Health). Researchers who want access to data sets from MoBa for replication should apply to www.helsedata.no/en. Access to data sets requires approval from The Regional Committee for Medical and Health Research Ethics in Norway and an agreement with MoBa. Data from the GraviD cohort cannot be made freely available as they are subject to secrecy in accordance with the Swedish Public Access to Information and Secrecy Act [Offentlighets- och sekretesslagen, OSL, 2009:400], but can be made available to researchers upon request. Access to data requires approval from the Swedish Ethical Review Authority (https://etikprovningsmyndigheten.se). Requests for data should be made to gravid@gu.se.

## Ethics statement

The studies involving human participants were reviewed and approved by The Swedish Ethical Review Authority, The Regional Ethics Committee in Gothenburg, and The Regional Committee for Medical and Health Research Ethics in Northern Norway. Written informed consent to participate in this study was provided by all participating women and from both parents for the child’s participation.

## Author contributions

HA initiated the study. HA, LB, AA, EP, AB, LL, HM, and AW planned the study. HA and AB is responsible for data protection and access. AA conducted the statistical analyzes and wrote the first version of the manuscript. HA, LB, and EP assisted with the statistical analyzes. All authors contributed to the article and approved the submitted version.

## Funding

The Norwegian Mother, Father and Child Cohort Study is supported by the Norwegian Ministry of Health and Care Services and the Ministry of Education and Research. The Norwegian Institute of Public Health has contributed to funding of the Norwegian Environmental Biobank. The GraviD cohort study was funded by the Swedish Research Council for Health, Working Life and Welfare (HA: 2012-0793 and 2018-00441), the Regional Research and Development grants (HA: VGFOUREG-388201 and VGFOUREG-229331) and the Sahlgrenska Academy.

## Conflict of interest

The authors declare that the research was conducted in the absence of any commercial or financial relationships that could be construed as a potential conflict of interest.

## Publisher’s note

All claims expressed in this article are solely those of the authors and do not necessarily represent those of their affiliated organizations, or those of the publisher, the editors and the reviewers. Any product that may be evaluated in this article, or claim that may be made by its manufacturer, is not guaranteed or endorsed by the publisher.
