# Supplementary material for: Maternal vitamin D status and risk of childhood overweight at 5 years of age in two Nordic cohort studies
Source: Front Nutr. 2023 Jul 26;10:1201171. doi: 10.3389/fnut.2023.1201171 (PMC10410266; doi:10.3389/fnut.2023.1201171)
Supplement: Supplementary file 1 [file Data_Sheet_1.docx]

Supplementary Material

Maternal vitamin D status and risk of childhood overweight at 5 years of age in two Nordic cohort studies

**Anna Amberntsson*, Linnea Bärebring, Anna Winkvist, Lauren Lissner, Helle Margrete Meltzer, Anne Lise Brantsæter, Eleni Papadopoulou, Hanna Augustin**

*** Correspondence:** Anna Amberntsson: anna.amberntsson@gu.se





**Supplementary Figure 1.** Sample selection from the MoBa sub-cohort and the GraviD cohort to the final study populations.

**Supplementary Table 1.** Retention and anthropometric measures of the children at the 11 ages in the MoBa sub-cohort (N=2,744).

| **Intended age** | **Reported age** | **Weight (kg)** | | **Response rate** | **Height (cm)** | | **Response rate** |
| --- | --- | --- | --- | --- | --- | --- | --- |
|  | **Mean (SD)** | **N** | **Mean (SD)** | **%** | **N** | **Mean (SD)** | **%** |
| 1.5 months | 1.5 (0.2) | 2457 | 5.1 (0.7) | 90 | 1983 | 57 (2) | 72 |
| 3 months | 3.1 (0.3) | 2659 | 6.4 (0.8) | 97 | 2614 | 62 (2) | 95 |
| 6 months | 5.9 (0.4) | 2572 | 8.0 (1.0) | 94 | 2549 | 68 (2) | 93 |
| 8 months | 8.1 (0.8) | 2499 | 8.8 (1.1) | 91 | 2489 | 71 (3) | 91 |
| 12 months | 12.0 (0.6) | 2500 | 9.9 (1.2) | 91 | 2498 | 77 (3) | 91 |
| 15–18 months | 15.9 (1.2) | 2506 | 10.9 (1.9) | 91 | 2497 | 81 (3) | 91 |
| 2 years | 2.1 (0.2) | 1595 | 13.0 (1.5) | 58 | 1606 | 89 (4) | 59 |
| 3 years | 3.0 (0.1) | 2317 | 15.1 (1.7) | 84 | 2262 | 97 (4) | 82 |
| 5 years | 5.2 (0.3) | 1944 | 20.0 (2.8) | 71 | 1958 | 113 (5) | 71 |
| 7 years | 7.1 (0.1) | 1970 | 25.0 (4.0) | 72 | 2008 | 126 (7) | 73 |
| 8 years | 8.1 (0.1) | 1339 | 28.3 (4.6) | 49 | 1381 | 132 (6) | 50 |

**
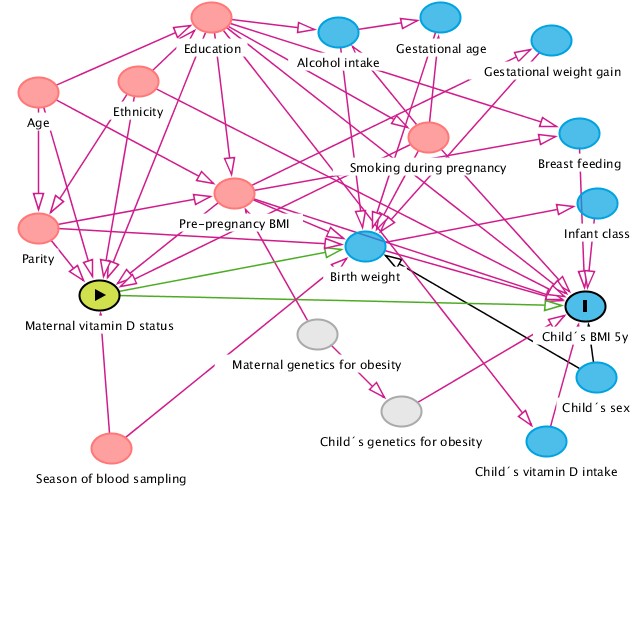
**

**Supplementary Figure 2.** A directed acyclic graph for the association between maternal 25OHD and child´s BMI at 5 years of age. Abbreviations: 25OHD, 25-hydroxyvitamin D; BMI, Body Mass Index

**
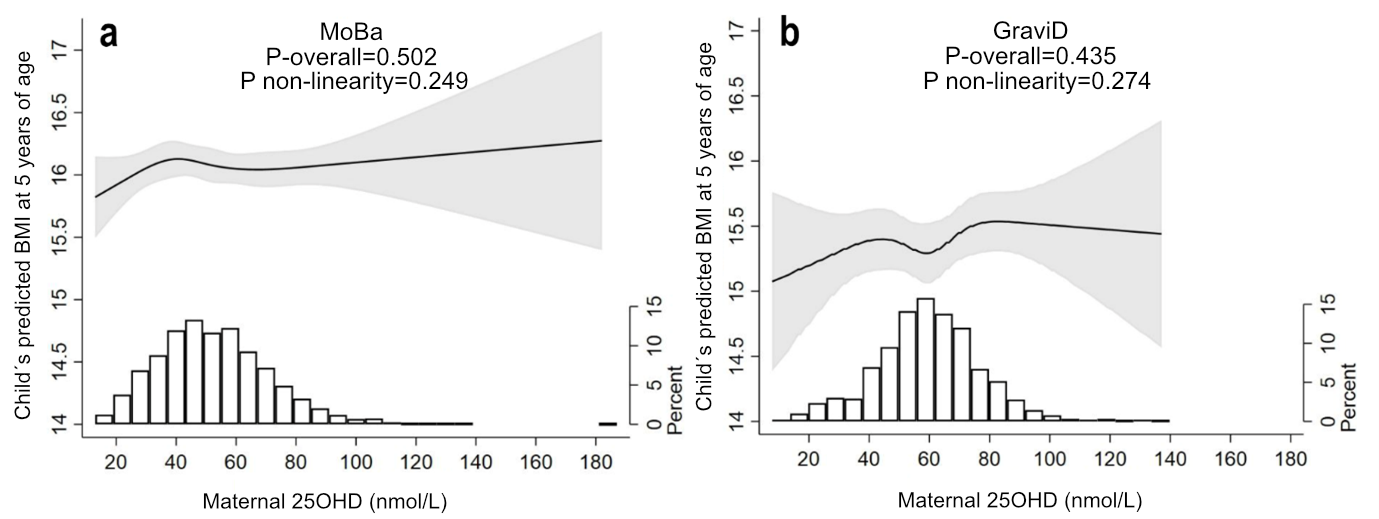

Supplementary Figure 3.** The association between maternal 25OHD and child´s BMI at 5 years of age in a) the MoBa sub-cohort (N=2,744), and b) the GraviD cohort (N=891). Knots were located at 24, 40, 51, 62 and 85 nmol/L in MoBa, and 29, 50, 60, 70 and 89 nmol/L in GraviD in the linear regression model, adjusted for maternal education, country of origin, pre-pregnancy BMI, child´s sex, maternal age, smoking during pregnancy, and parity. Abbreviations: 25OHD, 25-hydroxyvitamin D; BMI, Body Mass Index

**
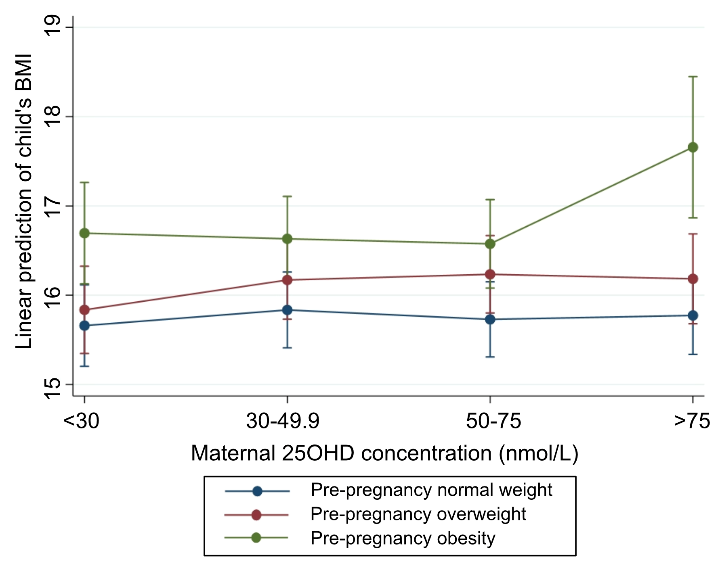
**

**Supplementary Figure 4.** The association between maternal 25OHD and child´s BMI at 5 years of age by maternal pre-pregnancy BMI in the pooled dataset (N=3,635). Including maternal education, country of origin, child´s sex, maternal age, smoking during pregnancy, and parity in a mixed effects linear regression model with cohort as random effect. For children of women with pre-pregnancy normal weight, no significant association was observed between maternal 25OHD and child´s BMI at 5 years of age (50-75 nmol/L: β=-0.04, 95% CI -0.19 to 0.11, p=0.598; 30-49.9 nmol/L: β=0.07, 95% CI -0.08 to 0.23, p=0.355; <30 nmol/L: β=-0.09, 95% CI -0.31 to 0.14, p=0.446). For children of mothers with pre-pregnancy overweight, maternal 25OHD 50-75 nmol/L, 30-49.9, and <30 nmol/L was associated with lower child´s BMI at 5 years of age compared with maternal 25OHD >75 nmol/L (β=-0.02, 95% CI -0.36 to 0.33, p=0.920; β=-0.11, 95% CI -0.46 to 0.25, p=0.562; β=-0.48, 95% CI -0.91 to -0.05, p=0.029). For children of mothers with pre-pregnancy obesity, maternal 25OHD 50-75 nmol/L, 30-49.9, and <30 nmol/L was associated with lower child´s BMI at 5 years of age compared with maternal 25OHD >75 nmol/L (β=-1.03, 95% CI -1.96 to -0.11, p=0.029; β=-0.98, 95% CI -1.89 to -0.06, p=0.036; β=-0.89, 95% CI -1.90 to 0.13, p=0.086). Abbreviations: 25OHD, 25-hydroxyvitamin D; BMI, Body Mass Index

**
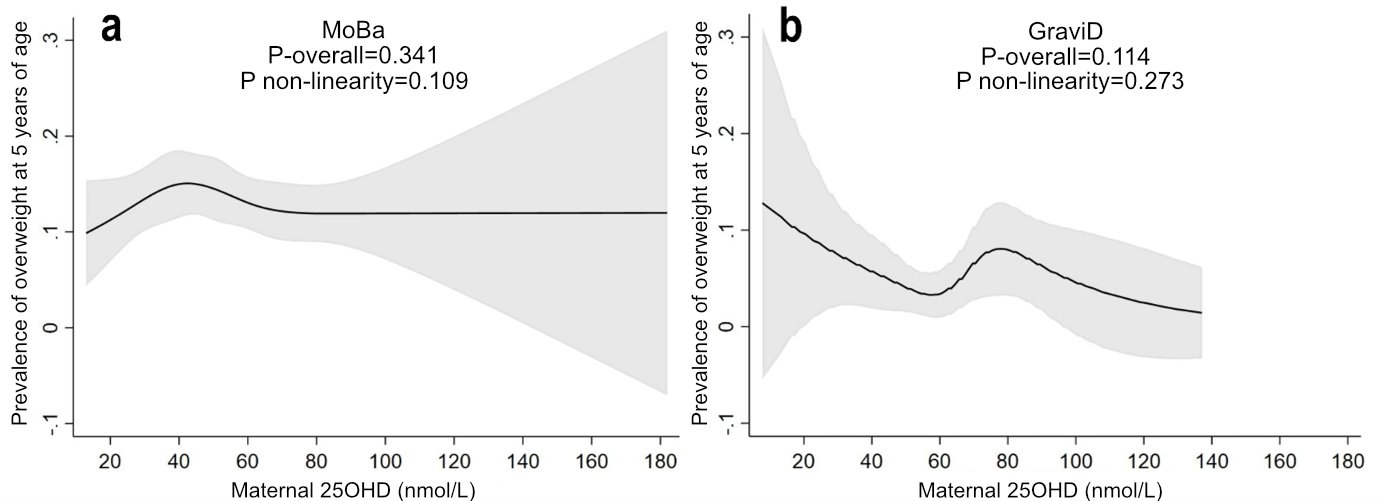

Supplementary Figure 5.** The association between maternal 25OHD and the prevalence of childhood overweight at 5 years of age in a) the MoBa sub-cohort (N=2,744), and b) the GraviD cohort (N=891). Knots were located at 24, 40, 51, 62 and 85 nmol/L in MoBa, and 29, 50, 60, 70 and 89 nmol/L in GraviD in the logistic regression model, adjusted for maternal education, country of origin, pre-pregnancy BMI, child´s sex, maternal age, smoking during pregnancy, and parity. Abbreviations: 25OHD, 25-hydroxyvitamin D.

**
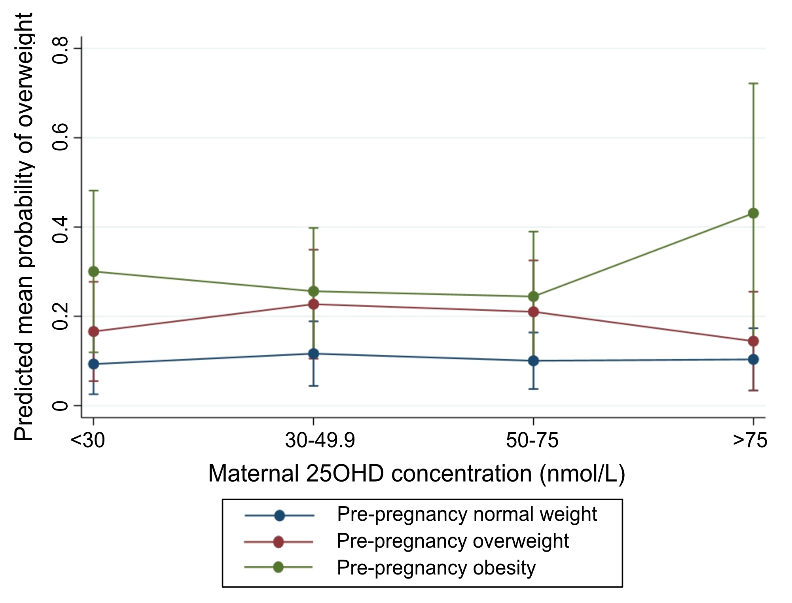
**

**Supplementary Figure 6.** The association between maternal 25OHD and the child’s probability of overweight at 5 years of age by category of pre-pregnancy BMI in the pooled dataset (N=3,635). Including maternal education, country of origin, child´s sex, maternal age, smoking during pregnancy, and parity in a mixed effects logistic regression model with cohort as random effect. Effect estimates in: children of mothers with normal weight (50-75 nmol/L: OR 0.96, 95% CI 0.66-1.40, p=0.832; 30-49.9 nmol/L: OR=1.15, 95% CI 0.78-1.70, p=0.475; <30 nmol/L: OR=0.89, 95% CI 0.51-1.55, p=0.681), children of mothers with pre-pregnancy overweight (50-75 nmol/L: OR 1.50, 95% CI 0.77-2.94, p=0.237; 30-49.9 nmol/L: OR=1.67, 95% CI 0.84-3.29, p=0.143; <30 nmol/L: OR=1.06, 95% CI 0.48-2.35, p=0.887), children of mothers with pre-pregnancy obesity (50-75 nmol/L: OR 0.47, 95% CI 0.14-1.58, p=0.221; 30-49.9 nmol/L: OR=0.54, 95% CI 0.16-1.79, p=0.314; <30 nmol/L: OR=0.70, 95% CI 0.19-2.60, p=0.594). Abbreviations: 25OHD, 25-hydroxyvitamin; BMI, Body Mass Index
